# Chronic Cutaneus Mucormycosis in an Immunocompetent Female

**DOI:** 10.5812/ircmj.1953

**Published:** 2013-03-05

**Authors:** Ali Akbar Heydari, Abdolhamid Fata, Maryam Mojtabavi

**Affiliations:** 1 Imam Reza Hospital. Mashad University of Medical Sciences, Mashad, IR Iran.

**Keywords:** Cutaneous, Infection, Mucormycosis, Patients

## Abstract

**Introduction:**

Cutaneous infection is an uncommon presentation of mucormycosis, usually seen after trauma, at the site of surgical drains or after occlusive dressings. The involved area is erythematous and painful, with varying degree of central necrosis. We report the case of chronic coetaneous infection of one year duration and without apparent necrosis in an immunocompetant patient.

**Case presentation:**

A 32-year-old immunocompetent woman presented with a large unilateral firm infiltrative plaque resembling cancer lesions, disfiguring the eyelids, nose and lips. The punch biopsy and then surgical debridement was done and the diagnosis of cutaneous mucormycosis was confirmed on histologic examinations with granulomatous reaction and characteristic broad, nonseptate, pale-staining hyphae.

**Conclusions:**

Mucomycosis should be in differncial diagnosis of any chronic infiltrative lesions even without visible necrosis and normal immune status of the patient.

## 1. Introduction

Cutaneous mucormycosis has been related to contaminated elastic bandages as a distinct entity (1, 2) but has been reported after intramuscular injection, minor trauma and contamination of devitalized tissue during major traumatic accidents. Burn wounds and insect bite can result in cutaneous mucormycosis. Our patients presented with refractory cellulites without any clear immunodeficiency.

## 2. Case Presentation

**Figure 1.**
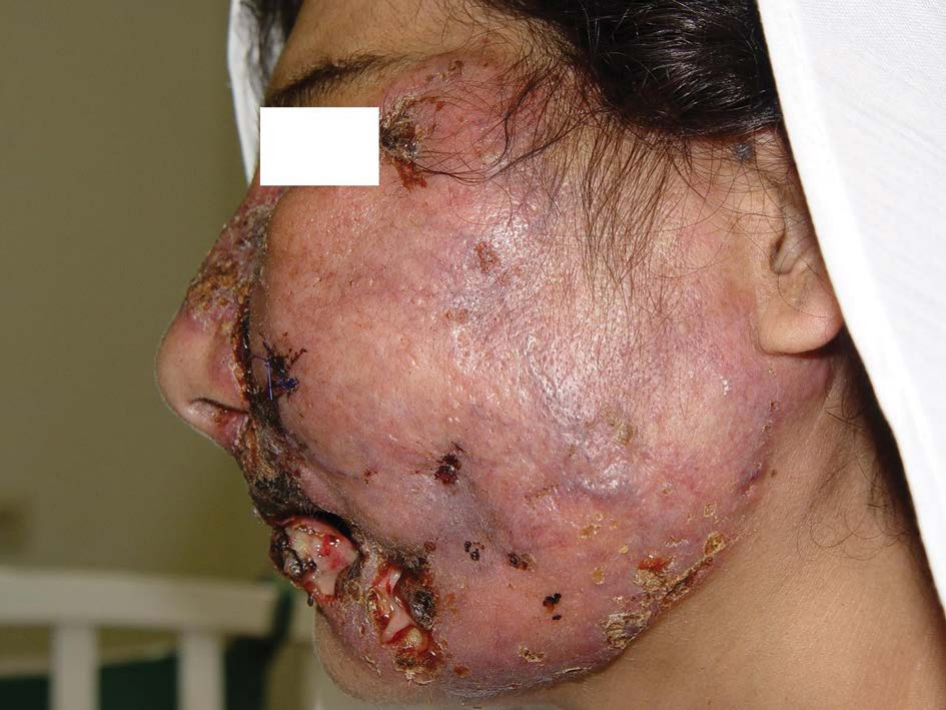
Chronic Cutaneus Mucormycosisin an Immunocompetent Female

A 32-year-old immunocompetent woman without any history of medical or surgical diseases, referred to our clinic, with unilateral edematous plaque on the left side of the face involving cheek, eyelids, nose and lips, with some ulcers which had purulent exudates on them (Figure 1).

The lesion had begun from 18 months ago. The lesion persisted till 5-6 months ago with no cure. Since then, the surrounded skin gradually became red, erythematous, and other ulcers appeared on the skin. On physical examination, the left side of the face was edematous and red and some ulcers with purulent exudates were seen. The lesion involved the nose and the lips but nasal and buccal mucosa was intact. CT-scanning of paranasal sinuses showed soft tissue thickness of the left cheek without involvement of the ipsilateral sinuses. Evaluations on exudate specimens revealed branched and aseptate hyphae, indicating mucormycosis. Punch biopsy and then surgical biopsy were done and both showed granulomatos dermatitis accompanying broad branched and aseptate hyphae that invaded the vascular endothelium. These findings confirmed the diagnosis of mucormycosis and therapy with Amphotricin-B deoxycholate was begun. After the patient became stable, extensive repetitive debridement of the involved area were performed. Therapy with amphotricine-B deoxycholate continued for a total dose of 2 g with good outcome and recovery of the lesion. The patient was then referred for plastic surgery.

## 3. Discussion

Mucormycosis is a rare human infection and may result of decreased efficacy of human immune system. Cutaneous infections with these organisms show a great deal of variability. Necrotic and hemorrhagic lesions with black scars or gangrenous cellulitis may be seen. Erythema is often noted surrounding the lesions. Erythematous papules, nodules, plaques without superficial necrosis, shallow ulcerations and chronic infections that persisted for months or years have also been described (3). Also there are a few reports of chronic primary coutaneus mucormycosis especially in immunocompetent females with clinical presentation of a slowly enlarging, erythematous plaque with slight elevated, circinate borders (4, 5). Our patient presented with similar scenario, but she had a firm, woody and infiltrative plaque which leads us to misdiagnosis of malignancy. The presence of angioivasion and absence of hypha encasement by eosinophilia material ( Splenore-Hoeppli material) in hematoxylin and eosin -stained tissue section differentiated mucomycosis from the related zygomycetes, Entomophtorals. The diagnosis of zygomycosis is easily made on tissue section. Involved tissue demonstrates focal areas of infection and may appear nodular or may produce extensive areas of necrosis with accompanying hemorrhage into the tissue. Invasion of the blood vessels (angioinvasion) by hyphal elements is generally seen in infections with the mucorals. The hallmark of a zygomycosis includes the demonstration of wide, ribbon-like, hyaline, predominantly aseptate hyphae with wide-angle branching. Surgical resection, amphotericin B (1 mg/kg per day IV) and reversal of any underlying medical conditions are the mainstays of therapy.
